# CML patients in the molecular era – report of five years experience of diagnosis and treatment in a single center

**Published:** 2013-09-25

**Authors:** I Voican, AM Vladareanu, H Bumbea, L Barsan, D Vasile

**Affiliations:** Department of Hematology, University Hospital Bucharest, Romania

**Keywords:** chronic myeloid leukemia (CML), molecular analysis, polymerase-chain-reaction (PCR), national registry

## Abstract

Chronic myeloid leukemia (CML) represents about 15% of all leukemia cases. Although the incidence of the disease is rather low, the therapeutic progress of the last decade has dramatically changed the evolution of this disease, whose survival considerably increased and in whom we now speak even about cure. The success of the therapy is strongly connected to the precocity of the diagnosis and molecular targeted therapy that implies a close monitoring of the patient. The specific molecular assay, that developed a lot in the last years, became an important tool in the management of these patients, providing the possibility of efficient changing in therapy. The purpose of our study was to identify the characteristics of our CML patients in terms of clinical and biological behavior. We analyzed 21 patients diagnosed between October 2007 and December 2010 and compared the data with a historical group of patients, also diagnosed in our department between March 2005 and September 2007. We found a better outcome and overall survival in the study group, due to improved diagnosis and monitoring techniques as well as to better access to therapy.

Abbreviations

accelerated phase (AP), blast phase (BP), Chronic myeloid leukemia (CML), chronic phase (CP), complete cytogenetic response (CCyR), complete hematologic response (CHR), complete molecular response (CMolR), European LeukemiaNet (ELN), Imatinib mesylate (IM) , major molecular response (MMolR), minimal cytogenetic response (minCyR), partial cytogenetic response (PCyR), polymerase-chain-reaction (PCR), Qualitative Polymerase-Chain-Reaction (Q-PCR), Quantitative Real-Time Polymerase-Chain-Reaction (RT-PCR), tyrosine kinase inhibitors (TKI)

## Introduction

Chronic myelogenous leukemia (CML) has been recognized as a clinical entity since the early 19th century. It is the first disease cytogenetically characterized by clonal expansion of hematopoietic cells carrying the Philadelphia chromosome. This is the result of a reciprocal translocation between the long arms of chromosomes 9 and 22, which generates BCR-ABL fusion gene that encodes a chimeric protein with strong tyrosine kinase activity and an important role in cell division.

 In 1998, a molecule able to inhibit the activity of BCR ABL fusion gene, Imatinib mesylate (IM) opened a new era in the therapy of the patients diagnosed with chronic myeloid leukemia, and thus created new perspectives in the treatment of malignancies in general. IM is the first example of targeted gene therapy in malignant disease [**1,2**].

 With the advent of tyrosine kinase inhibitors (TKI), it became mandatory to find more accurate techniques of assessing therapeutic response, necessary to guide the therapy, because on the one hand, pharmacological advances led to the discovery of second generation, more potent TKI, and on the other hand, monitoring patients treated with IM showed that although most people who received treatment since the early chronic phase achieved good responses, there is still a significant proportion of patients in whom treatment fails, either due to resistance or intolerance to treatment [**3**]. 

 In recent years, along with the generally accepted use of TKI as first-line therapy for CML patients, new therapeutic and monitoring guidelines were developed [**4,5**], which required clear and consistent criteria to define treatment response. Currently, the monitoring of modern therapy for patients with CML requires not only clinical and hematologic monitoring but, most importantly, periodic assessment of the cytogenetic and molecular response. It was shown that on the one hand, there is a definite correlation between the reduction in the number of leukemic cells (assessed by cytogenetic and molecular response) and the probability of progression to advanced stages of the disease and, on the other hand, it is important to early identify those patients who do not respond or lose optimal response, in order to offer them other effective treatment modalities [**6,7**], available today. Once the patient has achieved a complete cytogenetic response (CCR), the most sensitive method of measuring residual disease is monitoring BCR-ABL transcript by Quantitative Real-Time Polymerase-Chain-Reaction (RT-Q-PCR).

 Reduced BCR-ABL transcript levels correlate closely with a lower percentage of Ph positive metaphases, but the test is really valuable for patients who have already obtained CCR. Thus, when a 2 log reduction of the transcript is achieved, cytogenetic assay identifies no Ph positive cells and a 3 log reduction of transcript defines a major molecular response (MMR), when the number of residual leukemic cells is of about 106 - 107 [**8**]. Common techniques do not manage to identify the transcript, when it decreases over 4-5 logs. As a rule, the cytogenetic examination of the bone marrow should be performed at diagnosis, at 3, 6, 12 and 18 months and annually thereafter, as well as in all cases of treatment failure or any unexplained cytopenia. Molecular monitoring should be performed at every 3 months after starting the treatment with TKI until obtaining MMR and at least at every six months thereafter. 

 Although molecular assay remains the most useful method of monitoring patients with CCR (as well as rare cases of Ph negative CML), further cytogenetic tests retains value, as they can reveal other abnormalities in Ph cells -negative or signs of myelodysplasia. 

 Therapeutic advances in the field of chronic myeloid leukemia have required the development of more sensitive methods of monitoring disease evolution and response to treatment. Comparing the data provided by different laboratories, clear criteria have been established for the standardization of the results, so that each laboratory can use the international scale which means adopting a conversion factor specific to each laboratory [**9,10**]. 

## Objectives

 Our study was developed as part of a larger national project, "MONITORIZAREA MOLECULARA A ONCOGENEI HIBRID BCR-ABL IN LEUCEMIA MIELOIDA CRONICA (LMC) PRIN PCR REAL TIME CANTITATIV: FONDAREA REGISTRULUI NATIONAL PENTRU PACIENTII CU LMC" / "MOLECULAR MONITORING OF HYBRID BCR-ABL ONCOGENE IN CHRONIC MYELOGENOUS LEUKEMIA (CML) THROUGH CANTITATIVE REAL TIME PCR: FOUNDATION OF NATIONAL REGISTRY FOR CML PATIENTS", developed between October 2007 - December 2010, which included four university clinics in the country, under the leadership of "Carol Davila" University of Medicine and Pharmacy and the Hematology Department of Fundeni Clinical Institute.

 In the following paper, we will present the results obtained in our group of patients diagnosed in the Hematology Department of the University Emergency Hospital Bucharest.

 The aims of this study were: 

 a. Identification and analysis of molecular abnormalities in patients with CML. 

 b. Monitoring of BCR-ABL molecular transcript by RT-Q-PCR according to disease stage and treatment model.

 c. Founding the National Registry of patients with CML 

## Materials and method

Our study had two directions: the identification of new cases of CML who were treated and monitored in our clinic between October 2007 and December 2010 (prospective study) and a retrospective analysis of the cases diagnosed between March 2005 - September 2007 in the light of the new European LeukemiaNet (ELN) criteria for monitoring the treatment with TKI (retrospective study).

 The prospective study included 21 patients. Diagnosis was established according to WHO criteria for CML in the three phases of the disease: chronic phase (CP), accelerated phase (AP) and blast phase (BP). General information included patient assessment: a family history of neoplastic diseases (including hemato-oncological disease), conditions of life and work (social and economic status, educational level, rural / urban residence, exposure to toxic substances, smoking), clinical data (presence of general symptoms, WHO performance status assessment, splenomegaly and extramedullary determinations). Several specific parameters were followed: biological parameters (blood count with differential blood count detailed, liver and kidney function tests), bone marrow aspirate (morphological examination was performed in our Hematology Laboratory), bone marrow biopsy (histological examination performed at Victor Babes Institute). Cytogenetic examination of bone marrow aspirates was performed in Fundeni Clinical Institute and Victor Babes Institute (chromosome banding analysis + / - FISH) and molecular analysis of peripheral blood samples by qualitative PCR and RT-Q-PCR was done by the molecular biology laboratory of Fundeni Clinical Institute. It was possible to analyze the mutational status by sequencing of the ABL kinase domain for a very small number of patients in the molecular biology laboratory of Fundeni Clinical Institute. Patients were followed according to the ELN recommendations, in terms of hematologic, cytogenetic and molecular response at 3, 6, 12 and 18 months after starting TKI and whenever it was needed at other time points. We present below the definition of the response (**Table 1**) and the new criteria used for the evaluation of the response to IM (**Table 2**), as recommended by the ELN experts group – 2010.

**Table 1:** Response definition to Imatinib therapy according to ELN 2010

Risk
Hematologic response:
Complete (CHR):
WBC count < 10 x 109/L
Platelet count < 450 x 109/L
Differential: no immature granulocytes, <5% basophiles
No signs/ symptoms related
Non enlarged spleen
Cytogenetic response:
Complete (CCR): no Ph+ metaphases
Partial (PCR): 1 – 35% Ph+ metaphases
Minor (mCR): 36 – 65% Ph+ metaphases
Minimal (minCR): 66 – 95% Ph+ metaphases
None (noCR): > 95% Ph+ metaphases
Molecular response:
Complete (CMR): undetectable BCR-ABL mRNA transcripts by RT-Q-PCR in two consecutives blood samples
Major (MMR): BCR-ABL/ABL < 0,1% on the international scale

**Table 2:** Definition of response to Imatinib treatment 400mg daily for chronic phase CML patients

Time	Optimal response	Suboptimal response	Failure	Warnings
Diagnosis	Not applicable	Not applicable	Not applicable	High risk CCA in Ph+ clone*
3 months	CHR or at least mCR	No CR	Less than CHR	Not applicable
6 months	At least PCR	Less than PCR	No CR	Not applicable
12 months	CCR	PCR	Less than PCR	Less than MMR
Any time during treatment	Stable or improving MMR	Loss of MMR Mutations still sensitive to IM	Loss of CHR Loss of CCR Mutations poorly sensitive to TKI CCA in Ph+ clone*	Increase in transcript levels CCA in Ph- clone
* Identification of chromosomal abnormalities in Ph + cells represents a warning factor at diagnosis, but their appearance during treatment suggests clonal progression and treatment failure. Two consecutive tests are needed to show the same cytogenetic chromosomal abnormalities in at least two Ph + cells.

 The retrospective study included patients diagnosed with CML between March 2005 and September 2007 whose medical records were reviewed in light of the new ELN recommendations, both in terms of diagnosis (according to WHO criteria) and TKI therapy results (according to ELN criteria described above). We believe that the systematization of this data will contribute to the development of the National Registry database, which will provide a more complete perspective on the real situation of this disease in Romania. 

## Results

 1. 21 CML patients were diagnosed between October 2007 and December 2010, as it follows:

 • 2007: 4 patients 

 • 2008: 3 patients

 • 2009: 5 patients 

 • 2010: 9 patients

 Among these patients, 14 could be evaluated at 3 months, 12 patients - at 6 months, 8 patients - at 12 months and 6 patients at 18 months. The remaining patients who entered the study were not evaluated at the time points mentioned above, due to the loss of follow-up (1 patient) or to early death (2 patients).

 2. Distribution according to the demographic parameters group:

 • Sex: male / female: 12 / 9

 • The average age at diagnosis (min - max): 48.28 years (21-70)

 • Residence: urban 52.38%, rural 47.61% 

 3. Disease stage at diagnosis:

 • CP: 76.19%

 • AP: 9.52%

 • BP: 14.28% 

 4. Splenomegaly (long axis by ultrasound measurement > 120mm and palpable spleen) at diagnosis: 76.19%

 5. Hematological abnormalities in peripheral blood at diagnosis:

 • Anemia (Hb <12 g/dL): 57.14% 

 • Leukocytosis 50 – 100 x 109/L = 9,52%

 100- 200 x 109/L = 57,14%

 >200 x 109/L = 28,57%

 • Thrombocytosis (T> 450 x 109/L) = 28.57%

 6. Symptomatic at diagnosis (fever, weight loss, abdominal pain, bone pain, fatigue, sweating, infection, bleeding syndrome, etc.): 61.90% 

 7. 16 patients were evaluated for the Sokal index (only those in chronic phase at diagnosis). The distribution according to the risk category was as follows: 

 • Low risk: 6 / 16 (37.5%) 

 • Intermediate Risk: 5 / 16 (31.25%) 

 • High risk: 5 / 16 (18.75%) 

 8. Only two out of 21 patients presented other chromosomal abnormalities at diagnosis (9.52%). Both patients had high Sokal score. 

 9. Nine patients had values of BCR-ABL transcript exceeding 100% at diagnosis (42,85%). 

 10. Fifteen patients out of 21 patients diagnosed, were treated with IM, and 14 of these were evaluated at 3 months (one patient was lost to follow-up before 3 months of diagnosis), 12 at 6 months, 8 at 12 months and 6 at 18 months.

 • 3 months assessment: performed as recommended by the ELN:

 - complete hematologic response (CHR): 85.72% had CHR at 3 months

 - cytogenetic examination could be performed only in 3 patients, two of them obtaining partial cytogenetic response (PCR) (note that cytogenetic evaluation at 3 months is a recent recommendation ELN expert, so it was applied only for patients diagnosed in the second half of 2010)

 - molecular response at 3 months for 11 evaluable patients: no reduction - 1 patient (9.09%), 1 log reduction - 6 patients (54,54%), 2 log reduction - 3 patients (27.27%), more than 3 log reduction - 1 patient (9.09%)

 • 6 months assessment: only 11 out of the 14 patients initially treated with IM were evaluated at this time because: 1 patient was switched to Nilotinib due to Imatinb intolerance and two other patients had less than 6 months of treatment until the present evaluation. The results were as it follows:

 - cytogenetic response: CCR - 3 patients (27.27%), PCR- 5 patients (45.45%), minor cytogenetic response (mCR) – 1 patient (9.09%), minimal cytogenetic response (minCR) - 0 patients (0%), no response - 2 patients (18.18%)

 - molecular response (reduction of the transcript related to diagnosis): no reduction - 3 patients (27.27%), 1 log reduction - 2 patients (18.18%), 2 log reduction - 2 patients (18.18%), > 3 log reduction - 4 patients (36.36%). We should mention that only 8 patients were analyzed both at 3 and 6 months, because some patients either did not have 6 months of treatment with IM or they were switched to the 2nd generation TKI. Six of these patients had a significant decrease in transcript level (at least 1 log) before the 3 months evaluation, 1 patient had a constant level of transcript and in 1 patient, the transcript level increased, consistently with the loss of CHR and lack of any cytogenetic response (considered treatment failure according to ELN criteria). Consequently, this patient received treatment with second generation TKI (Dasatinib)

 • 12 months assessment: 7 patients were evaluated for cytogenetic response (1 sample without mitosis) and 7 patients for molecular response 

 - cytogenetic response: CCR - 5 patients, PCR: - 1 patient, minCR – 1 patient, no response - 0 patients

 - molecular response (reduction of the transcript related to diagnosis): 1 log reduction - 1 patient, 2 log reduction - 1 patient, > 3 log reduction - 4 patients. Conclusion of the evaluation at 12 months: 3 patients had MMR and 1 patient had complete molecular response (CMR)

 • 18 months assessment: only 14 patients out of 14 treated with TKI were evaluated at 18 months: 

 - cytogenetic response: CCR - 5 patients, PCR – 1 patient

 - molecular response: CMR - 3 patients (1 patient maintained the previous response, 2 patients obtained CMR, reported to the previous assessment), MMR - 2 patients, 1 log reduction- 1 patient (who obtained only PCR)

 11. Correlation of cytogenetic response with molecular response 

 We comparatively analyzed the cytogenetic and molecular responses at 6, 12 and 18 months. Given that systematic examination of cytogenetic response at 3 months after starting treatment with TKI is a very recent recommendation of the ELN expert group, only a small subset of patients diagnosed in 2010 were subjected to a cytogenetic evaluation at this time point, so this interval was not analyzed.

 - Diagnosis analysis: 19 patients who were evaluated for cytogenetic and molecular response. The number of Ph positive metaphases was between 14 and 100% and the transcript level was between 27 and 132.9%.

 There was no correlation between the percentage of positive metaphases and the transcript in this subset of patients. 

 - 6 months analysis: 10 patients could be analyzed in terms of both cytogenetic and molecular response: 

 • no cytogenetic response (2 patients)- no reduction or a 1 log reduction of the transcript (both patients were diagnosed in BP) 

 • mCR (1 patient) - 1 log reduction of the transcript

 • PCR (4 patients) – 1 to 4 log reduction

 • CCR (3 patients) – 3 to 4 log reduction

 In this subset of patients we observed a good correlation between the type of the cytogenetic response and the degree of the molecular response. 

 - 12 months analysis : 7 patients were evaluated both for molecular and cytogenetic response

 • no cytogenetic response (1 patient) - 1 log transcript reduction (patient diagnosed in BP and converted to CP, by using TKI treatment)

 • mCCR (1 patient) – 1 log transcript reduction ( the same response from the previous assessment)

 • PCR (1 patient) – 4 log transcript reduction

 • CCR (4 patients) - at least 3 log decrease of transcript (3 patients with MMR and one patient with CMR)

 In this subset of patients there was a better correlation between the type of cytogenetic response and the degree of the molecular response, meaning that patients who had obtained CCR also had a transcript reduction for at least 3 log. The only discordance was observed in patients with PCR, who had an unexpectedly low transcript level. We initially thought that this result might be due to the poor quality of the sample, but further examination showed CCR and also MMR, which led us to the conclusion that the patient was actually a "late responder".

 - 18 months analysis: 6 patients were evaluated

 • 1 patient with PCR - transcript reduction by 2 log (but without achieving MMR) 

 • 5 patients CCR - transcript reduction by at least 3 log (3 patients in this subgroup obtaining MMR and 1 patient CMR).

 There was a good correlation between the cytogenetic and molecular response in this subgroup of patients, which suggests that significant decrease in tumor mass, makes the RT-Q-PCR to become a useful tool for treatment monitoring. 

 12. Correlations between cytogenetic and molecular response and evolution under treatment

 In the subgroup of patients monitored for at least 18 months, we have noticed the following: 

 • One patient did not achieve CCR. The best cytogenetic response was PCR (at 18 months) and best molecular response was 2-log transcript reduction, which means less than MMR (considered the optimal response at 18 months). However, both the molecular and cytogenetic response was continuously improved during treatment, although slow and suboptimal. CHR was maintained constant and the quality of life of the patient was still very good. 

 • One patient achieved late CCR (at 18 months) and MMR and this response was not sustained during the next monitoring period, which justified switching to a 2nd generation TKI; with this therapy, a new CCR, as well as MMR was quickly obtained (after 3 months of treatment with the 2nd generation TKI). 

 • 3 patients achieved CCR after 6 months: in all three patients we observed a 1-2 log reduction of the transcript level at 3 months evaluation, a 2-4 log reduction at 6 months, and a decrease of at least 4 log at 12 months evaluation, so that at 12 months, 2 patients had MMR and 1 patient had CMR, and at 18 months, 1 patient had MMR and 2 patients had CMR. We can presume that the early achievement of CCR creates premises for an optimal early molecular response, of a better quality and perhaps even for healing (for patients with CMR). 

 13. The evolution to more advanced stages was not observed in any of the 16 patients diagnosed in CP. Of the two patients diagnosed in AP, one progressed rapidly (<1 month) to BP and died shortly after, the other was converted in CP and this phase still lasts. Of the three patients diagnosed with BP, one died after a 16-month evolution, the other two patients are currently alive under treatment. 

 14. Survival at 24 months in the group of patients diagnosed between October 2007 - December 2008 was the following: 7 patients were diagnosed, but one patient was lost at the 3 months follow-up. Six patients out of the initial 7 patients were diagnosed in CP and 1 patient in BP. The survival for the BP patient was of 16 months, which exceeded by far the rate of survival of a blast crisis treated with conventional chemotherapy. The other five patients, all diagnosed in CP, survived for over 24 months (practically all patients are alive at the moment of this analysis). The survival of patients diagnosed before the beginning of this study was analyzed for comparison, meaning during March 2005 - December 2006: of the five patients diagnosed during this period, four were evaluable for comparison (one patient lost to follow-up before 24 months); 2 patients were diagnosed in CP and two in AP, all patients were treated with IM after more than six months from the diagnosis, only two patients survived for more than 24 months (including 1 patient initially diagnosed in accelerated phase). The conclusion is that survival at 24 months was better in the group of patients included in our study than in the previous group, due to the early initiation of treatment with TKI and the possibility of a better monitoring of treatment response, which allowed an early modulation of medication (escalating doses of IM and access to 2nd generation TKI) depending on the evolution of molecular response.

 15. Evolution correlated to Sokal index:

 We analyzed the response to Imatinib at 3, 6, 12 and 18 months correlated with Sokal index values (**Table 3**)

 In conclusion, in our study group:

 • No major differences were noted in the evolution of patients with low and intermediate Sokal index - all these patients achieved an optimal response at 3, 6, 12 and 18 months although 1 patient ("late responder") lost CCR after more than 18 months follow-up, but achieved a new optimal response to the 2nd generation TKI. 

 • All patients with high Sokal index had an optimal response (CHR) at 3 months, but then the optimal response rate decreased; however, even the patient who never achieved CCR, maintained the CHR and PCR after more than 18 months of treatment.

**Table 3:** Therapy response related to prognostic value of Sokal Index

Sokal Index	3 months	6 months	12 months	18 months	Comments
Low = 3 pts	CHR – 3 pts	CCR – 2 pts PCR – 1 pts	CCR – 2 pts PCR – 1 pt	<CCR – 3 pts MMR – 2 pts CMR – 1 pt	Loss of CMR and switch to 2nd generation TKI - 1 pt
	Optimal response – 3 pts	Optimal response – 3 pts	Optimal response – 2 pts	Optimal response – 3 pts	
Intermediate = 4 pts	CHR – 4 pts	CCR – 1 pt PCR – 3 pts	CCR – 2 pts	CCR -2 pts CMR – 2 pts	2 pts followed-up at 12 and 18 months
	Optimal Response – 4 pts	Optimal response – 4 pts	Optimal response – 2 pts	Optimal response – 2 pts	
High = 2 pts	CHR – 2 pts	PCR – 1 pt mCR – 1 pt	CCR – 1 pt mCR – 1pt	1 pt at 18 months follow- up– no molecular response, but maintains CHR and PCR	
Optimal response - 2 pts	Optimal response – 1pt	Optimal response – 1 pt			

## Results

 The analysis of our study group, that included 21 patients diagnosed with CML in October 2007 - December 2010, revealed the following:

 1. The reported incidence of CML in SUUB Hematology Department is small (21 newly diagnosed cases reported between 2007-2010 of about 2400 cases of hemato-oncology disorders diagnosed during the same period), which matches with the relatively low incidence of the disease in the general population. 

 2. Staging of CML patients from our study may reflect the specificity of our department that functions in an emergency hospital: the majority of cases were symptomatic at diagnosis, some with hyperleukocytosis, anemia or impressive splenomegaly and quite a large number of patients were diagnosed in advanced stages of disease: 6 patients with advanced disease out of a total of 27 patients diagnosed with CML in our clinic from 2005 until now.

 3. The use of standard monitoring parameters, according to a strict schedule, as recommended by ELN guidelines, enabled the comparison of the results obtained after an efficient treatment and the adjustment of therapy, thus providing the basics to obtain a maximum benefit, given that the modern treatment of CML is costly to society but proved highly beneficial for the patient. Therefore, a careful monitoring of therapy with all means available at this time and in all time points of a schedule, it is absolutely essential because only in this way we can early identify those patients with low chances to get the optimal response to a certain therapy line. These patients can now benefit from other effective treatment options.

 4. Optimal response to treatment correlated both with the precocity of diagnosis (patients with low risk factors, diagnosed in early stage disease obtained more quickly both the CCR and the MMR) and with the earliness of initiation of TKI therapy: thus, in the patients group diagnosed and treated from 2007 to 2010, who started Imatinib therapy early after diagnosis, there was no case of progression to more advanced stages of the disease compared to patients diagnosed before this time and who often received treatment with TKI more than six months after diagnosis. Furthermore, among patients diagnosed in advanced stages of disease (5 patients with AP or BP) there were only two deaths (2 out of 3 patients diagnosed BP) and of these, the only one patient treated with TKI, had a 16-month survival.

## Conclusions

 The TKI that dramatically changed the evolution and prognosis of CML, also raised new problems in managing these patients, because of the need for accurate and careful monitoring of therapy in order to identify, as early as possible, the non-responders or those patients who acquire resistance to drugs and who may benefit from other treatment modalities if these changes are identified in time. This is the direction in which the experts’ efforts are now focused, technical progress in terms of therapy and monitoring of the disease at the molecular level, being actually interconnected. CML is probably offering the most successful model of co-operation of the top experts in the field of molecular biology with those in the pharmaceutical, and ultimately is the proof of the enormous benefit for the patient that brings the implementation of these innovations in current practice.

 The development of the National CML Registry is an urgent requirement for the Romanian integration in European and international medicine. Knowledge of the disease in the population of our country will enable the identification of epidemiological peculiarities of the disease in this geographical area, will allow the classification of each case in specific risk groups, based on standardized criteria, will provide patient access to best therapy, specific to each stage of this disease and, on the long-term, will enable the improvement of the diagnostic and therapeutic modalities in accordance with international standards.

Acknowledgements: This articles is part of a larger national grant – PNCD I research Project no 041-087/2007: "Molecular monitoring of hybrid BCR-ABL oncogene in chronic myelogenous leukemia (CML) through cantitative real time PCR: foundation of national registry for CML patients" (2007 – 2010).

 The authors would like to thank the following colleagues for their contribution to the entire grant and to the current article: Madalina Begu MD, Mihaela Gaman MD (Department of Hematology, University Hospital Bucharest, Romania), D Coriu MD, Rodica Talmaci MD, Cerasela Jardan MD, Camelia Dobrea MD, Daniela Iancu MD (Department of Hematology, Fundeni Clinical Institute, Bucharest, Romania), Anca Ilea MD (Ritus Biotec Laboratory, Codlea, Romania), Aurora Arghir MD ("Victor Babes" National Institute of Pathology, Bucharest, Romania)
